# Age and extraversion differences in heart rate reactivity during working memory tasks

**DOI:** 10.1371/journal.pone.0245539

**Published:** 2021-01-22

**Authors:** Ann Pearman, Shevaun D. Neupert, Gilda E. Ennis

**Affiliations:** 1 School of Psychology, Georgia Institute of Technology, Atlanta, Georgia, United States of America; 2 Department of Psychology, North Carolina State University, Raleigh, North Carolina, United States of America; 3 Wisconsin Alzheimer’s Disease Research Center, University of Wisconsin, Madison, Wisconsin, United States of America; University of Colorado Denver, UNITED STATES

## Abstract

Research and theory have shown a link between heart rate reactivity during cognitive testing and extraversion in younger adults; however, similar work has not been conducted with older adults. This study was designed to explore age and extraversion-related differences in within-person heart rate (HR) reactivity during two working memory tasks of varying difficulty using a multi-level modeling approach. Across 570 total within-person assessments of continuous HR monitoring, 28 younger adults (*M* = 19.76, *SD* = 1.15) and 29 older adults (*M* = 71.19, *SD* = 6.63) were administered two working memory tasks (backward digit span and *n*-back). There were no age differences in reactivity during the backward digit span. However, similar to previous findings, on the more difficult *n*-back task, younger adults low in extraversion showed a trend toward higher HR reactivity than young adults high in extraversion. Interestingly, the older adults showed the opposite pattern in that lower extraversion older adults were less reactive than the higher extraversion older adults who showed the steepest increase in HR. The HR increase of the older adults high in extraversion may be an indication of higher engagement in this more difficult task. Individual differences in extraversion need to be taken into account when administering working memory tasks in older adults.

## Introduction

Extraversion is the personality trait most associated with sociability, excitability, and emotional expressiveness [1, 2]. Adults high in extraversion tend to endorse items related to engagement with the outside world, social activity, and assertiveness. Eysenck (1955) first suggested that people low in extraversion may be more sensitive to social interactions because a lower threshold for autonomic nervous system arousal makes them more physiologically reactive than people high in extraversion [3]. These findings have been both confirmed and extended [4–8] since those initial studies. For instance, following a study of a large group of undergraduates under conditions of social stress, Hinton and Craske [8] concluded that “extraverts have stronger parasympathetic control relative to introverts” (p. 27). These reactivity differences do not appear to be limited to social stressors. Jonassaint and colleagues [4] found that higher extraversion was related to lower cardiovascular reactivity, as measured by various measures of cardiac output to both a mental stress task and an emotional stressor in a group of young and middle-age adults (ages 19–51 years). More recently, Lü and colleagues reported that this extraversion-related reactivity to stressors also varied by stressor intensity in a sample of young adults such that participants low in extraversion were less reactive to moderately stressful social situations but were more reactive to high intensity situations [7]. On the whole, young adults low in extraversion do tend to show heightened cardiovascular reactivity (i.e. changes in heart rate in response to stimuli) to mild-to-moderate laboratory stressors compared to younger adults high in extraversion.

While there is a scarcity of similar research on the link between extraversion and cardiovascular reactivity in older adults, there has been a preponderance of work in the past 10 years examining more general cardiovascular reactivity in this population (e.g. [9–12]). Although older adults, on average, have lower resting mean heart rate (HR) than younger adults, age differences in HR reactivity are less clear. Uchino and colleagues [12] conducted a meta-analysis on studies examining age differences in HR reactivity. They found that, overall, older adults had lower HR responses to laboratory-based stressors than younger adults. However, what we do not know from these studies is whether there is variability in this HR response by extraversion (or by task). Hess (2014) suggested that when faced with certain types of challenging tasks, such as cognitive tests, older adults may need to expend more cardiovascular effort [13].

In line with this hypothesis, we propose that cognitive challenges, such as working memory tasks of varying difficulty, may elicit differential responses from younger and older adult with older adults having a greater response on the more difficult task. In addition, given the differential relationship of extraversion on many psychological variables in young and older adults, we had reason to believe that the extraversion–heart rate reactivity relationship may also show different patterns in older adults than is commonly seen in younger adults. In the current study, we use working memory tasks as stressors to examine these relationships. Working memory tasks were chosen because they have been shown to elicit differential cardiovascular responses by extraversion level in young and middle-aged adults [5, 14–16]. For instance, Fink et al. found that on several working memory tasks participants low on extraversion showed more event-related desynchronization in line with as tasks became more difficult. Understanding the age differences in the effect of extraversion on functioning may provide researchers and clinicians with an enhanced way to understand individual differences in how older adults respond physically to cognitive challenges.

### The current study

The current study was designed to examine differential within-task heart rate response to working memory tests in terms of age and extraversion. Specifically, we examined HR trajectories during two working memory tasks of different difficulty levels (a digit span task and the *n*-back) in terms of extraversion using an extreme age-groups design (younger vs. older adults).

Using a multilevel modeling procedure, we aimed to examine within-task reactivity differences between a sample of younger and older adults. We predicted that the more difficult working memory task (*n*-back) would be associated with more HR reactivity compared to the less difficult task (backward digit span) within each group with older adults showing slightly higher reactivity. In addition, we predicted that participants lower in extraversion would show higher HR reactivity than participants higher in extraversion. However, given the lack of studies examining extraversion and HR reactivity in older adults, we did not make specific predictions about the direction of potential age differences in reactivity or the effects of extraversion-reactivity on performance.

## Method

### Participants

University undergraduates were recruited through flyers and classroom announcements, and community-dwelling older adults were recruited through local advertisements and a participant database. The older adult participant database is a commonly used recruitment tool in studies of community dwelling older adults. Volunteers were excluded if they met any of the following self-reported criteria: stroke in the last five years, serious head injury, Parkinson’s disease, less than a high school education, subjective poor health, on prescription stimulants, or non-native English speakers. Participants were also screened for gross cognitive impairment using a modified-for-telephone version of the Short Portable Mental Status Questionnaire [17]. The final sample consisted of 29 younger adults (*M* = 19.76, *SD* = 1.15, range = 18–23 years, 68% female) and 31 older adults (*M* = 71.19, *SD* = 6.63, range = 60–85 years, 59% female). All participants were given a $25 honorarium at the completion of the study. The Brandeis University Institutional Review Board approved this study. Consent was obtained with a written in-person consent process and form.

The aims for the present study focus on within-person and within-task changes in reactivity, so power was primarily derived from the number of within-person assessments (*n* = 570 total, 285 for each task) [18]. We computed post hoc estimates of power [19] within the F test family for the most similar statistical test available: Repeated measures ANOVA containing within-subject and between-subject interactions. We had a power level of .82 when assuming a small effect size (.15), which is a conservative estimate of power because ANOVA-based models assume balanced data and compound symmetry whereas MLM is more flexible and powerful [20].

### Materials

#### Working memory tasks

The Backward Digit Span (BDS) from the Wechsler Adult Intelligence Scale–Third Edition (WAIS-III; [21]) and the *n*-back task [22] were orally administered and used as working memory measures. The BDS requires the participants to repeat back a series of numbers in reverse order and is considered the easier of the two task both due to length and subjective difficulty [23]. The test begins with two numbers, increasing until the participant commits two errors. High score for BDS is 8. The *n*-back requires participants to listen to a sequence of numbers and to indicate the number that was presented *n* trials beforehand. The present study used 1- and 2-back targets. A mean of the two trials was calculated for a combined score that can range from 0 to 30. A subtle but important difference between these two tasks is that the BDS is stopped when participants cannot progress further (i.e. two consecutive errors) but all of the *n*-back items are administered regardless of performance. The trials on the BDS were, therefore, of different lengths for each person whereas the trials of the *n*-back were approximately the same length.

#### Extraversion

The Midlife Development Inventory (MIDI) Personality Scale (MIDI; [24]) was used to assess personality. This scale has been shown to be highly stable [25] and is correlated with the longer NEO-FFI [26]. Because the scope of this paper was focused on expanding our understanding of age and extraversion differences in reactivity in response to stressors, only the extraversion scale was used here. The MIDI extraversion scale consists of five items asking participants to indicate how well each item describes them on a scale ranging from 1 (a lot) to 4 (not at all). Two of the five items represent the excitability facet of extraversion (i.e. active, lively), two of the five items represent the sociability facet of extraversion (i.e. friendly, talkative) and a last item (i.e. outgoing) straddles both facets of extraversion. Items were reverse coded when necessary so that higher scores indicate higher levels of extraversion. A composite extraversion score was calculated from the mean of these items. Cronbach’s alpha for the current sample was .78.

#### Subjective assessments

A subjective assessment of task difficulty was taken immediately following each working memory tasks. Participants were asked to respond about the perceived difficulty level on a 5-point scale ranging from 1 (low) and 5 (high).

#### Assessment of heart rate

Heart rate (HR) was continuously measured with a fingertip photoplethysomograph (PPG) sensor placed on the third finger of the participant’s non-dominant hand with a Velcro piece. The MEDAC System/3 is an integrated instrumentation and software package for non-invasive monitoring of a range of physiological measures from the autonomic nervous system. The MEDAC program takes measurements every 1/100^th^ of a second and calculates HR in beats/minute (bpm). We instructed all subjects to keep their hand and fingers still during the recording phase, and any movements were noted by the experimenter. The interviewer inserted a marker in the psychophysiological data when each new task began and ended. When conducting the analyses, we selected the data relevant to the marker of interest for each cognitive task. Aggregated assessments were obtained by dividing the task time into quintiles for each person and then taking the mean of the data points within each quintile. The quintiles were chosen and created so that the number of within-person observations would (1) be consistent across participants so as to be able to compare within person trajectories, regardless of the time it took persons to complete the task, and (2) to allow for the possibility of non-linear (quadratic) effects, and to capture possible differences in response due to the different ending points of the tasks (i.e. administration till completion vs. administration to failure). Thus, we obtained five timepoints for each of the working memory tasks, for a total of 280 assessments (5 timepoints * 2 tasks * 28 participants) for the younger adults and 290 assessments (5 timepoints * 2 tasks *29 participants) for the older adults. Because the trials on the BDS were of different lengths, this method allowed us to examine individualized trajectories of change that would be standard in number across participants, but would be flexible within participants to adjust for the rate of completion.

#### Covariates

Because many older adults are on medications to control hypertension and these medications can affect HR response [27], we included cardiovascular medications as a covariate in our analyses. Current medications were given by participants and were then coded for presence (1) or absence (0) of cardiovascular medications. Cardiovascular medications included angiotensin converting enzyme (ACE) inhibitors (e.g., lisinopril), calcium channel blockers (e.g., veraprinil), and beta blockers (e.g., atenolol). Seventy-four percent of the older adult sample was on one or more hypertension medications. None of the younger adults were on hypertension medication. While participants were encouraged to not drink caffeine or smoke cigarettes for two hours prior to the session, we did not record reports of use of these substances.

### Procedure

Participants were tested individually in a laboratory room by trained research assistants. After written informed consent was obtained, physiological sensors were attached to the participants’ non-dominant hand. All participants were given a brief period (approximately 5 minutes) to acclimate to the testing room environment and equipment before the recording was started. Following this stabilization period, HR was continuously recorded for the entire session which lasted approximately an hour. Participants did several non-cognitive tasks (e.g. filling out questionnaires) prior to beginning the cognitive battery to allow for further acclimation to the testing setting. The entire acclimation period lasted for approximately 20 minutes. The baseline HR mean was obtained from this time period. Following this acclimation period, participants were given a series of cognitive tasks as well as the subjective task questions following each task. The BDS was given near the beginning of the session (~25 minutes into recording), and the *n*-back task was in the middle of the session (~40 minutes into recording). At the conclusion of the testing, participants filled out several more questionnaires, including the personality measure.

### Analyses

Multilevel models [20] were conducted using Proc Mixed with SAS 9.4 [28] to examine age and extraversion differences in within-person HR trajectories during the working memory tasks. We used quintile-level heart rate as the dependent variable in each analysis.

A fully unconditional model indicated that 52% (τ_00_ = 90.39, *z* = 4.58, *p* < .001) of the variance in HR assessments was between people and 48% (σ^2^ = 82.96, *z* = 7.57, *p* < .001) was within people during the *n*-back task. For HR during the backward digit span test, 69% (τ_00_ = 94.19, *z* = 5.02, *p* < .001) of the variance was between people and 31% (σ^2^ = 42.02, *z* = 11.05, *p* < .001) was within people. Therefore, these two models indicated that there was significant variability in both levels for each of the dependent variables for further analyses.

At Level 1, each person’s variability (e.g., change in HR over time) is represented by an intercept and slope that become the outcome variables in a Level 2 model in which they may depend on person-level characteristics (e.g., age and extraversion). Time (quintile) was the Level 1 predictor and was operationalized as the reactivity slope. Baseline heart rate, blood pressure medication, performance on the cognitive task, age, extraversion, and Age X Extraversion were used as Level 2 predictors of the intercept. Age, extraversion, and Age X Extraversion were used as Level 2 predictors of the reactivity slope.

Specifically, the following model was used to test our hypothesis.

Level1:HRit=β0it+β1it(TIME)+rit (1)

Level2:β0i=γ00+γ01(BASELINEHR)+γ02(BLOODPRESSUREMED)+γ03(PERFORMANCE)+γ04(AGE)+γ05(EXTRAVERSION)+γ06(AGE*EXTRAVERSION)+u0i (2)

β1i=γ10+γ11(AGE)+γ12(EXTRAVERSION)+γ13(AGE*EXTRAVERSION)+u1i (3)

In Eq 1, the intercept (β_0it_) is defined as the expected level of HR for person i at the baseline HR assessment (i.e., TIME = 0). The slope (β_1it_) is the expected within-person change in HR across quintiles, and is operationalized as the reactivity slope. The error term (r_it_) represents a unique effect associated with person i (i.e., fluctuation around the mean). Eq 2 includes baseline heart rate, blood pressure medication, and task performance as covariates and tests for age and extraversion differences in the average level of HR. The intercept (γ_00_) represents the average level of HR after accounting for baseline heart rate, blood pressure medication, and performance on the task, age, and extraversion. Eq 3 tests for age and extraversion differences in the reactivity slope (i.e., the within-person association between time and HR), with the intercept (γ_10_) representing the average reactivity slope. Interindividual fluctuations from the average HR level are represented by u_0i_, and interindividual fluctuations from the reactivity slope are represented by u_1i_. Datasets can be found in S1 File.

## Results

### Descriptive analyses

Table 1 shows the means, standard deviations, and zero-order correlation coefficients of the study variables split by age group. There were no age differences in levels of extraversion. On the 1 to 5 difficulty scale, younger and older adults rated the BDS significantly less difficult than the *n*-back. The only extraversion–performance relationship was with younger adults on the BDS (*r* [26] = .48, *p* = .01) with high extraversion relating to better performance which is commonly found in younger adults [29]. For all other task and age-group combinations, there were no significant correlations between extraversion and performance. Extraversion was, however, negatively related to perceived digit span task difficulty for the older adults such that older adults high in extraversion reported the digit span as less difficult than older adults low in extraversion. Means of HR during the tasks are reported in Table 1 as well. As a group, older participants had slightly lower mean HR than younger participants. In addition, older adults had lower performance on the *n*-back than the younger adults but there were no age differences in HR on the task.

**Table 1:** Descriptive statistics and Pearson correlation coefficients for study variables split by age group.

Variable	Younger (n = 28)		Older (*n* = 29)									
	*M*	*sd*	*M*	*sd*	1.	2.	3.	4.	5.	6.	7.	8.
Demographics												
1. Age	19.79_a_	1.17	71.66_a_	6.61	--	**-.11**	**-.22**	**-.13**	**.01**	**.28**	**-.03**	**.02**
Personality												
2. Extraversion	3.53	.44	3.41	.58	-.25	--	.48*	**-.05**	**-.02**	**.07**	**-.01**	**-.09**
Performance												
3. Digit Span	5.61	1.37	5.14	1.48	-.24	.08**	--	**.11**	**.33**^‡^	**.14**	**-.11**	**-.09**
4. *n*-back	25.56_a_	3.19	18.61_a_	5.84	-.32^‡^	.00**	.40**	--	-.07**	-.55**	**.04**	**-.01**
Task difficulty ratings												
5. Digit span	2.71_b_	.90	2.31_b_	.97	-.07	-.39**	-.01**	-.07**	--	**.34**^‡^	**-.04**	**.03**
6. *n*-back	3.18_b_	1.16	3.55_b_	1.12	.13	-.21**	-.26**	-.32^‡^*	.40**	--	**.12**	**.08**
Heart rate mean (BPM)												
7. Digit span	81.42_a__b_	9.09	73.74_a__b_	10.27	-.55**	.24	.11	-.02	-.16	-.31	--	.86**
8. *n*-back	79.24_a__b_	10.11	71.65_a__b_	9.68	-.56**	.17	.10	-.04	-.23	-.29	.92**	--

*Note*. Younger adult correlation coefficients above the diagonal in bold and older adults below the diagonal. Task difficulty measured on a 1 to 5 scale with 5 being very difficult. BPM = beats per minute.

_a_Between-group age differences significant at *p* < .05.

_b_Within age-group differences significant at *p* < .05.

^‡^p ≤ .10

*p < .05

**p < .01

### HR reactivity during BDS

The conditional intercept reflecting the average heart rate adjusted for all predictors in the model was 9.03 (*t* = 0.61, *p* = .5546, 95%CI [-20.79, 38.85]), but there was a significant effect of baseline HR (γ_01_ = 0.88, *t* = 18.70, *p* < .0001, 95%CI [0.79, 0.98]). There was a non-significant change in HR during the BDS (γ_10_ = -4.74, *t* = -0.94, *p* = .35, 95%CI [-14.73, 5.24]). There were also no significant age differences (γ_11_ = 0.09, *t* = 1.02, *p* = .307, 95%CI [-0.08, 0.25]) or Extraversion X Age differences (γ_13_ = -0.02, *t* = -0.89, *p* = .372, 95%CI [-0.07, 0.03]) in rates of change. The effects were independent of performance on the BDS task (γ_03_ = 0.63, *t* = 1.74, *p* = .089, 95%CI [-0.10, 1.35]). There were significant individual differences around the reactivity slope (τ_11_ = 2.63, *z* = 2.32, *p* = .01), indicating that reactivity was not the same for all people. This model accounted for 74% of the between-person and 29% of the within-person variance in HR during the BDS. Estimates of variance explained were calculated using the pseudo R^2^ method outlined by Raudenbush and Bryk [20] (Level 1: [σ^2^uc—σ^2^c]/σ^2^uc; Level 2: [τ_00_uc—τ_00_c]/ τ_00_uc, with the reduction in variance from the unconditional (uc) to conditional (c) model divided by the unconditional variance).

### HR reactivity during the *n*-back

The conditional intercept reflecting the average heart rate adjusted for all predictors in the model was 0.08 (*t* < 0.01, *p* = .997, 95%CI [-44.95, 44.11]), but there was a significant effect of baseline HR (γ_01_ = 0.80, *t* = 8.40, *p* < .0001, 95%CI [0.61, 0.99]). There was a non-significant overall trend for changes in HR (γ_10_ = 10.65, *t* = 1.84, *p* = .07, 95%CI [-0.73, 22.03]) during the *n*-back. Unlike the BDS, however, there were significant age differences in rates of change (γ_11_ = -0.22, *t* = -2.29, *p* = .023, 95%CI [-0.40, -0.03]), and the age differences in rates of change were further moderated by extraversion (γ_13_ = 0.07, *t* = 2.43, *p* = .016, 95%CI [0.01, 0.12]). The effects were independent of blood pressure medication (γ_02_ = 1.87, *t* = 0.71, *p* = .478, 95%CI [-3.40, 7.15]) and performance on the *n*-back (γ_03_ = -0.17, *t* = -0.14, *p* = .892, 95%CI [-2.74, 2.39]). There was no variance around the reactivity slope between people so the slope was constrained to be equal across participants. This model accounted for 67% of the between-person and 19% of the within-person variability in HR during the *n*-back.

**Fig 1:**
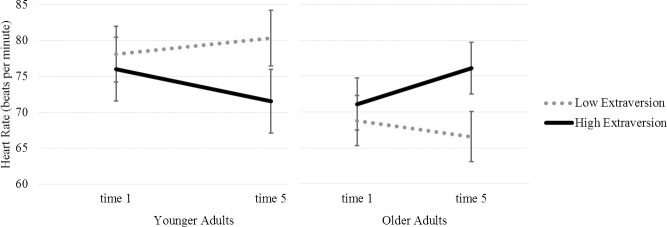
Predicted points and error bars for the time X age X extraversion interaction predicting heart rate during the *n*-back test. The error bars represent the 95% CI around the predicted points, and were obtained through separate estimate statements in SAS proc mixed for each of the points.

Separate analyses conducted by age group were conducted to decompose the 3-way interaction of Extraversion X Age X Time (Fig 1). Older adults who were high in extraversion (*M* +1*SD* = 3.94) were more reactive compared to older adults low in extraversion (*M* -1*SD* = 2.68) (γ_11_ = 1.44, *t* = 2.49, *p* = .014, 95%CI [0.29, 2.59]). Younger adults with low extraversion (*M* -1*SD* = 3.07) experienced a mild increase in HR and young adults high in extraversion (*M* +1*SD* = 3.95) experienced a decrease in HR, but these effects were not significantly different from one another (γ_11_ = -1.89, *t* = -1.27, *p* = .207, 95%CI [-4.84, 1.06]).

## Discussion

This study was designed to examine within-person HR change during two working memory tasks of varying difficulty by age group and extraversion. Similar to previous studies, we demonstrated that on the more difficult working memory task (i.e. *n*-back) younger adults low in extraversion increased in HR during while young adults high in extraversion decreased (e.g. [4, 5]). Older adults, however, showed the opposite pattern in that older adults low in extraversion showed significant HR declines during the *n*-back task while older adults high in extraversion showed significant increases. In fact, highly extraverted older adults showed the steepest increase in HR overall.

One possible explanation for these findings is differential effort variability. Hess (2014) suggested that older adults who are more invested in performing well on cognitive tasks are more likely to engage in those tasks [13]. Our results show that older adults exerted the same cardiovascular effort as younger adults during the *n*-back task and performed significantly worse. Thus, in line with SET [13], older adults may need to exert even more effort to reduce or eliminate the age differences in performance. There is literature suggesting that older adults who are high in extraversion are more actively engaged both cognitive and physically with their environments than older adults low in extraversion [30, 31] and rate themselves higher on self-efficacy [32]. If high extraversion older adults are highly engaged in and believe they have the capacity to succeed on difficult cognitive tasks like the *n*-back, then they may put forth more effort which would be commensurate with the higher levels of HR activation seen during the *n*-back compared to the BDS. Conversely, for the more introverted older adults, the *n*-back may have been difficult enough to reach their threshold for decreased reactivity. This would be also be consistent with idea that persons higher in extraversion stay engaged past the time that persons lower in extraversion are able to stay maximally engaged in a difficult task [5]. To test this hypothesis, future studies will need to include a wider range of cognitive tasks along with questions about achievement motivation so as to better disentangle task-difficulty from task-engagement. In addition, future studies could compare different types of non-cognitive stressors (e.g. Trier Social Stress Test [33]) to the *n*-back. This would provide a clearer picture as to whether the differences seen here are specific to cognitive tasks which may elicit a different type of reactivity in older adults than non-cognitive tasks.

Younger adults may have not been as reactive because the *1-* & *2*-back may not have been of sufficient difficulty to substantially engage them. In a study using a 1-, 2-, 3-, and 4-back, Kemper and colleagues did not detect an introversion-related decrease in reactivity in their sample of younger adults until the 3-back [5]. In the current study, we only administered the 1- and 2-back which limits our ability to draw further similar conclusions. In addition, we did not measure degree of effort, engagement, or coping on these tasks which makes it difficult to make clear distinctions between motivation/effort and other possibilities both in younger and older adults. That is, it is possible that older adults high in extraversion were more motivated and engaged in the *n*-back task and therefore showed effort-related HR acceleration. But, it is also possible that the older adults high in extraversion were more anxious about performing the task in front of the research assistant and thus showed the HR increases. We consider this explanation less likely based on the extensive literature that shows that anxiety and extraversion tend to be negatively, not positively, correlated [34, 35]. Subsequent studies are underway to disentangle these possibilities.

Although the primary interest in this study was in HR reactivity during tasks and not performance on those tasks, we did find that our observed patterns of reactivity were independent of performance, which was also unrelated to HR in both tasks. Given that extraversion has been shown to be related to performance in adults, these findings are unexpected [29, 36, 37]. However, we used a smaller sample and different measures than those previously reported. It is certainly possible that the tasks we used were not difficult enough to detect age by extraversion performance relationships. In addition, most of the findings regarding extraversion and cognition are related to simple speed-of-processing tasks and not more complicated working memory tasks.

Our results further extend previous findings of extraversion having variable effects on psychological variables, such as health and attention, at different points in the adult lifespan [29, 38, 39]. Specifically, extraversion may affect cardiovascular reactivity differently in younger compared to older adults. Gomez and colleagues [38] found that extraversion was a predictor of subjective well-being in young adults but this relationship was moderated by goal importance in older adults. More recent findings suggest that even within samples of older adults extraversion differences contribute to a variety of real-world consequences. For instance, in older adults high extraversion has been shown to be related to better health outcomes [40] and financial satisfaction [41] compared to lower extraversion. Our results suggest that high extraversion in older adults may also be beneficial within the context of stressful cognitive testing situations.

There are several clear limitations of this study. The first is the use of quintiles for examination of within-task reactivity. While quintiles on their own is not necessarily a problem, other work has used anywhere from 10-second intervals to 30-second increments (e.g. [42, 43]). The data were extracted in quintiles to allow for possible cubic and quadratic trends (none were found). In addition, quintiles were chosen to make sure that each participant had 5-measurements over the course of each task (due to the differing lengths of the BDS). This potential limitation, however, is also its strength in that we are able to examine within-task reactivity and were not consigned to reactivity being a posttest minus pretest mean. We did, however, control for baseline HR in the models which strengthened the results. A second limitation is the extreme age group design rather than a full adult lifespan model. While there are limitations with this type of design, including not treating age as continuous and potential lack of generalizability, extreme age group designs have the benefit of being cost-efficient and serving as a productive avenue for future studies [44]. Also, the use of undergraduate students as well as an older adult participant pool does limit the generalizability of these results. That is, there is a chance that the results might be different with a community-dwelling sample of younger adults as compared to a college sample as college students tend to be accustomed to being tested in this setting and may have developed some immunity or compensatory coping mechanisms for it. However, it is, again, a good starting point for future studies about this topic. Finally, the lack of counterbalancing on the administration of the two working memory tasks makes it difficult to say with definitiveness that there was not an order effect of administering the harder of the two tasks second.

## Conclusion

Given the previous lack of studies examining the heart rate reactivity–extraversion link in older adults, this study extends previous findings with younger adults and may help explain some of the individual differences we see in cardiovascular reactivity during cognitive performance in later life. A major advantage of this paper is that the use of continuous monitoring of heart rate and the use of 5 quintiles of observations dramatically increased the power to detect within-person effects of HR response. There appear to be systematic effects of personality on HR responses which are dependent on age. Specifically, we found differential effects of extraversion on HR reactivity in older and younger adults. Our findings suggest that older adults lower in extraversion may be at risk for effort-related decreases in cardiovascular reactivity and, possibly, cognitive functioning. That is, if less extraverted older adults are less likely or able to psychologically and physiologically engage in difficult cognitive tasks, then this could put them at a disadvantage in terms of long-term performance and also daily functioning compared to more extraverted older adults.

## Supporting information

S1 File Study data. The compressed file contains HR data from both WM tasks.
(CSV)

S2 File (CSV)
